# Effects of parent–child interaction therapy dosage on child and parent outcomes: differentiating child‐directed interaction and parent‐directed interaction session impacts in child welfare‐involved families

**DOI:** 10.1111/jcpp.70106

**Published:** 2026-02-03

**Authors:** Xiaolan Liao, Julia P. Ruggiero, David E. Bard, Adon F. G. Rosen, Elizabeth A. Skowron

**Affiliations:** ^1^ Department of Developmental/Behavioral Pediatrics The University of Oklahoma of Health Sciences Center Oklahoma City OK USA; ^2^ Department of Psychology University of Oregon Eugene OR USA; ^3^ College of Arts & Sciences Vanderbilt University Nashville TN USA; ^4^ Department of Human Development and Family Studies Pennsylvania State University University Park PA USA

**Keywords:** PCIT, treatment dosage, parenting, child welfare, RCT

## Abstract

**Background:**

Parent–child interaction therapy (PCIT) improves parenting and child behavior, yet little is known about how dosage of its two phases, warm relationship building focused child‐directed interaction (CDI) sessions and safe, effective discipline skills‐focused parent‐directed interaction (PDI) sessions, contributes to outcomes, particularly in child welfare‐involved families. Understanding these dose–response patterns can clarify the pathways through which PCIT produces change.

**Methods:**

In a sample of 204 child welfare families with young children, we examined the dose–response relationship between each PCIT phase and key intervention outcomes of positive and negative parenting skills and disruptive child behavior problems. We also used sequential mediation models to test time‐ordered intervention dosage effects (i.e., number of CDI sessions completed and subsequent number of PDI sessions completed) on the parent and child outcomes.

**Results:**

Sequential mediation models showed that the PCIT intervention exerted significant indirect effects on increased positive parenting skills and decreased negative parenting behaviors and child behavior problems through higher dosage of relationship‐enhancing CDI sessions followed by higher dosage of safe discipline‐focused PDI sessions. Further, CDI dosage interacted with PDI dosage to predict greater gains in positive parenting skills outcomes.

**Conclusions:**

These results contribute new insights into the pathways through which PCIT shapes outcomes in a sample of child welfare‐involved families. Findings also highlight the significant unique contribution that limit‐setting‐oriented PDI, a relatively understudied phase of PCIT, plays in enhancing positive parenting skills and mitigating child behavior problems.

## Introduction

Over 3 million children are referred into the child welfare system each year in the United States and experience significant material and psychosocial hardships that have profound effects on their development (U.S. Department of Health & Human Services, 2024; Felitti et al., 1998). Parents who come to the attention of the child welfare system may show low warmth and affection and harsh, inconsistent, or disengaged parenting (Khoury, Rajamani, Bureau, Easterbrooks, & Lyons‐Ruth, 2020; Plate et al., 2019; Wilson, Rack, Shi, & Norris, 2008). Exposure to experiences such as physical abuse and neglect, present for some children in the child welfare system, heightens children's risk for a host of negative behavioral health outcomes, with almost half of children in the child welfare system experiencing significant behavioral health problems (Burns et al., 2004; Garner et al., 2021; Lippard & Nemeroff, 2020; Lupien, McEwen, Gunnar, & Heim, 2009; Moffitt et al., 2011).

Links between parenting quality and children's behavioral outcomes are well‐established: Positive, responsive parenting and safe, effective child management practices foster children's prosocial behavior, whereas negative, aversive parenting impairs it (Gruhn & Compas, 2020; Morris, Criss, Silk, & Houltberg, 2017; Ryan, Martin, & Brooks‐Gunn, 2006). Caregiving relationships that help children feel emotionally safe and supported, coupled with developmentally aligned support for their autonomy, yield positive emotional and behavioral outcomes for children like decreased reactivity and increased self‐regulation (Bernier, Carlson, & Whipple, 2010; Bridgett, Burt, Edwards, & Deater‐Deckard, 2015; Lavi, Katz, Ozer, & Gross, 2019). Conversely, chronic exposure to caregiver anger and aggression steers children toward heightened arousal, vigilance to negative emotional cues in the environment, and dysregulation (Briggs‐Gowan et al., 2015; Cicchetti & Rogosch, 2009; Cipriano‐Essel, Skowron, Stifter, & Teti, 2013; Pollak, Messner, Kistler, & Cohn, 2009; Sætren, Augusti, & Hafstad, 2021). In the short term, these behavioral strategies may be beneficial: They allow children to stay alert and ready to respond to potentially threatening situations. In the long term, however, such heightened reactivity and vigilance makes it difficult for children to regulate their behavior in support of goal‐directed activities and prosocial interactions, instead amplifying their risk for negative behavioral outcomes (Bar‐Haim, Lamy, Pergamin, Bakermans‐Kranenburg, & van IJzendoorn, 2007; Burris, Buss, LoBue, Pérez‐Edgar, & Field, 2019; Kammermeier, Duran Perez, König, & Paulus, 2020; Pollak & Kistler, 2002). Fortunately, there is growing evidence that attachment‐ and behavior‐based parenting interventions can strengthen child welfare‐involved children's behavioral regulation skills (Katz et al., 2020; Korom et al., 2021; Lind, Bernard, Ross, & Dozier, 2014; Neville et al., 2013).

One such intervention is parent–child interaction therapy (PCIT). PCIT, like other empirically supported parenting programs (e.g., the Incredible Years Program, Parent Management Training – Oregon Model, and the Triple P‐Positive Parenting Program), is theoretically grounded in social learning theory (Sanders, Kirby, Tellegen, & Day, 2014), and is recognized as an effective behavioral parent training program for reducing disruptive behavior and increasing prosocial behavior in young children (Campbell, Hawes, Swan, Thomas, & Zimmer‐Gembeck, 2023; Eyberg et al., 2001; McNeil, Robinson, Wallace, Quetsch, & Highlander, 2019). The effects of PCIT are durable over time (Hood & Eyberg, 2003; Thomas & Zimmer‐Gembeck, 2007) and generalize to school and community settings (Funderburk et al., 1998; Lieneman, Girard, Quetsch, & McNeil, 2020; McNeil, Eyberg, Eisenstadt, NewComb, & Funderburk, 1991) and the behavior of untreated siblings, as well (Brestan, Eyberg, Boggs, & Algina, 1997). Initially identified as an evidence‐based treatment for families of children with externalizing disorders, PCIT has increasingly been applied to families with other primary concerns, including families involved in the child welfare system who are at risk for child maltreatment (Batzer, Berg, Godinet, & Stotzer, 2018; Family First Prevention Services [FFPSA] Clearinghouse, 2020; National Traumatic Stress Network, 2022). PCIT helps caregivers increase levels of warmth, acceptance, and safe, effective limit‐setting that encourage children's positive social–emotional development while supporting their age‐appropriate autonomy (Larzelere, Morris, & Harrist, 2013). Meta‐analyses indicate that PCIT effectively decreases harsh, inconsistent, and coercive parenting, strengthens positive parenting skills, and reduces maltreatment risk in child welfare‐involved families (e.g., Euser, Alink, Stoltenborgh, Bakermans‐Kranenburg, & van IJzendoorn, 2015; Kennedy, Kim, Tripodi, Brown, & Gowdy, 2016; Zhang, Gatzke‐Kopp, & Skowron, 2024). Rigorous randomized clinical trials (RCTs) of PCIT for child welfare families conducted in the U.S. and Australia show that it lowers child abuse recidivism rates substantially (Chaffin et al., 2004; Chaffin, Funderburk, Bard, Valle, & Gurwitch, 2011), and reduces aversive parenting, strengthens positive parenting, and boosts parent self‐regulation skills (Skowron et al., 2024; Thomas & Zimmer‐Gembeck, 2011, 2012). For child welfare‐involved families, positive outcomes are observed in children as well, with RCT studies documenting reductions in child internalizing and externalizing problems when contrasted to a waitlist (Thomas & Zimmer‐Gembeck, 2011, 2012) or services‐as‐usual (SAU) (Skowron et al., 2024) controls. Likewise, randomized field trials of group‐based PCIT for foster‐care families have documented reductions in child welfare‐involved children's externalizing and internalizing problems relative to child welfare SAU (Mersky, Topitzes, Grant‐Savela, Brondino, & McNeil, 2016; Mersky, Topitzes, Janczewski, & McNeil, 2015), and studies using pre‐post designs have shown reductions in child aggression following PCIT services (Herschell, Scudder, Schaffner, & Slagel, 2017; Timmer, Urquiza, Zebell, & McGrath, 2005; Timmer, Ware, Urquiza, & Zebell, 2010). Further, PCIT is cost‐effective for child maltreatment prevention and is well‐supported for preventing out‐of‐home placements for children (Lee et al., 2012; Lee, Aos, & Miller, 2008; Title IV‐E Prevention Services Clearinghouse, 2019). PCIT is also effective with ethnically diverse families (e.g., Fernandez & Eyberg, 2009; Leung, Tsang, Sin, & Choi, 2015; Matos, Bauermeister, & Bernal, 2009; McCabe & Yeh, 2009).

### Parent–child interaction therapy

PCIT is typically conducted in clinic‐based sessions, where caregivers interact with their child in one room and receive live coaching via wireless earpiece from their therapist, who typically observes the session from a separate room (Funderburk & Eyberg, 2010; Zisser & Eyberg, 2010). By coaching from behind the scenes, therapists allow families to interact naturally, enabling children to perceive their caregivers as the primary agents of positive change (Skowron & Funderburk, 2022). PCIT is structured into two treatment phases: child‐directed interaction (CDI) and parent‐directed interaction (PDI). Each phase focuses on developing a unique set of caregiver skills theorized to contribute meaningfully to family outcomes. The goal of this research was to understand dose–response relationships between each PCIT treatment phase and key parenting skills and child behavior outcomes.

#### Child‐directed interaction sessions

All PCIT‐involved families begin with the CDI phase of treatment. CDI sessions are focused on relationship building: parents receive coaching on a small set of positive parenting skills (i.e., PRIDE skills: labeled Praise, Reflection, Imitation, Behavior Descriptions, and Enjoyment) designed to strengthen a parent's warm bond with their child, reinforce a child's good behavior, and increase the child's felt sense of safety (Funderburk & Eyberg, 2010). To ensure these early sessions maintain focus on building warmth between parent and child, therapists coach parents to refrain from using harsh, aversive parenting and actions that control the direction of the play (i.e., ‘Don't’ skills: commanding, questioning, and criticizing). Therapists gently block negative parenting in the moment by suggesting positive alternatives in real time. Therapists also teach caregivers to ignore children's minor misbehavior, a strategy that aligns with techniques used in other evidence‐based parenting programs (e.g., parent management training, Kazdin, 1997). By removing attention from minor misbehaviors, parents avoid inadvertently reinforcing their child's actions, allowing them to focus their attention on more desirable child behaviors instead (Skowron & Funderburk, 2022). Behavioral parenting interventions that are similar to PCIT but focus largely on developing the positive parenting skills like those found in the CDI phase of treatment (e.g., the Triple P‐Positive Parenting Program; Sanders, 1999) show short‐ and long‐term improvements in children's social, emotional, and behavioral health (Sanders et al., 2014; Sanders, Cann, & Markie‐Dadds, 2003).

Evidence supports the theoretical underpinnings of this first, relationship‐building phase of PCIT. Studies examining trajectories of change in PCIT suggest that some gains in parenting skills and child behaviors appear early on in treatment. For example, in Chaffin et al.'s (2004) randomized clinical trial, 70% of reductions in child welfare‐involved caregivers' negative parenting and improvements in positive parenting were observed by the fourth session of PCIT (Hakman, Chaffin, Funderburk, & Silovsky, 2009). Similarly, significant reductions in child behavior problems emerged with as few as four CDI phase sessions of PCIT in a naturalistic study of state‐wide PCIT implementation (Lieneman, Quetsch, Theodorou, Newton, & McNeil, 2019). The notable changes in family behavioral outcomes observed in this first, relationship‐building phase of PCIT suggest the skills introduced in these earliest sessions emphasizing positive parenting may provide families with essential building blocks for improving parenting skills and children's behavior outcomes.

#### Parent‐directed interaction sessions

Upon completion of the warm relationship‐building CDI phase, families transition into its second stage of PCIT treatment: PDI. These subsequent sessions help caregivers learn safe, effective limit‐setting skills to implement with their child. In PDI, therapists coach parents to use developmentally appropriate commands and to implement a consistent, predictable time‐out protocol in the event that children do not cooperate. With support from the PCIT therapist, parents learn to be consistent, predictable, and calm as they implement PDI with their child, following a series of steps that aim to dissipate the parent's emotional reaction and provide the child with predictable consequences to reinforce their child's prosocial responses (i.e., rule‐following, minding & listening). In between, parents are coached to transition back to use of positive parenting (i.e., PRIDE) skills learned in the first phase of PCIT (Eyberg & Funderburk, 2011).

Research on behavioral parenting programs that incorporate evidence‐based techniques for child behavior management (e.g., PCIT, Parenting Management Training, the Incredible Years) shows that positive discipline strategies like safe, effective limit‐setting and time‐out from positive reinforcement predict improvements in child behavioral outcomes and observed parenting skills (Dadds & Tully, 2019; Kaminski & Claussen, 2017; Kaminski, Valle, Filene, & Boyle, 2008; Larzelere, Gunnoe, Roberts, & Ferguson, 2016). When these limit‐setting strategies are combined with reinforcement of children's prosocial behavior, treatment effects are further enhanced (Kazdin, 1997). Limit‐setting focused sessions like those in PDI may be of particular relevance to child welfare‐involved families because most episodes of child physical abuse occur within a parent–child compliance interaction that becomes increasingly coercive, where harsh parental discipline techniques may be temporarily effective and reinforced by short‐term child compliance but harmful to children (Batzer et al., 2018; Chaffin et al., 2004). The positive discipline skills learned in PDI sessions may help to combat this cycle by helping parents learn to effectively utilize safe, consistent limit‐setting techniques that promote child cooperation and achieve compliance, while ensuring the child's felt sense of safety (Skowron & Funderburk, 2022).

While the PDI limit‐setting phase of PCIT has been theorized to be of unique importance for family outcomes, studies to date have rarely focused directly on this phase of treatment. One exception is a small‐scale trial that reversed the standard PCIT treatment order for half the study sample (e.g., *n* = 12 families received PDI first followed by CDI sessions) while half completed the standard order PCIT (*n* = 12 received CDI followed by PDI sessions), and reported greater improvements in child compliance and reductions in disruptive behaviors among families who received PDI sessions first (Eisenstadt, Eyberg, McNeil, NewComb, & Funderburk, 1993). Several other studies assessed progress at mid‐treatment (i.e., post‐CDI) and again at posttreatment (i.e., post‐PDI), allowing for an examination of the impact of PDI phase sessions on outcomes. For instance, although Jent et al. (2023) found that ~63% of children with clinically elevated behavior problems had scores within normal limits at mid‐treatment, that figure rose to nearly 90% of children with healthy behavior outcomes by posttreatment, demonstrating the additional benefits that accrue following PDI phase sessions. Similarly, consistent with the distinct targets of each phase of PCIT, Ramos et al. (2025) found that while caregivers tend to show the greatest improvements in positive parenting following the CDI phase, most families demonstrate continued reductions in child behavior problems during the PDI phase. Further, parent‐reported reductions in children's emotional lability (Lieneman et al., 2020) and dysregulation (Rothenberg, Weinstein, Dandes, Jent, & McNeil, 2019) have been observed after both CDI and PDI phases of treatment. Taken together, these studies suggest that PDI phase work focused on safe, consistent limit‐setting and positive reinforcement for child cooperation may facilitate important gains in children's behavioral outcomes.

### Skill threshold‐based versus time‐limited parent–child interaction therapy

Studies of PCIT have investigated the efficacy of both the standard, time‐unlimited PCIT and more recent time‐limited approaches. In most outpatient settings, true to the original treatment model, PCIT is delivered using a skills‐threshold‐based, time‐unlimited approach, where families continue in each phase of treatment until they meet standard parenting skill thresholds, with attrition defined as discontinuing treatment at any point before meeting the defined skills thresholds (Eyberg & Funderburk, 2011; Fernandez & Eyberg, 2009). As such, skills threshold‐based PCIT treatment can become protracted, presenting challenges as families who require additional sessions to meet CDI skill benchmarks experience delays in gaining access to PDI‐focused child management skill training. Many families involved in the child welfare system also contend with numerous stressors and resource limitations that can make regular attendance in lengthy treatments a challenge (Batzer et al., 2018; Skoranski et al., 2021). For families at increased risk for early termination, brief interventions with clear behavioral foci can be more effective than lengthier ones for increasing positive, responsive parenting skills in families facing these types of challenges (Bakermans‐Kranenburg, van IJzendoorn, & Juffer, 2003; Lanier et al., 2011; Timmer, Hawk, Usacheva, & Urquiza, 2021). To date, RCTs of PCIT with child welfare‐involved families have utilized session‐capped (i.e., time‐limited) protocols that provide families with some maximum number of CDI phase and PDI sessions in treatment when benchmarks are not yet achieved. Notably, studies have shown that time‐limited PCIT can be effective in reducing maltreatment recidivism and improving children's behavioral problems even among families who fail to meet PCIT skills thresholds (e.g., Chaffin et al., 2004; Skowron et al., 2024). In a head‐to‐head comparison of time‐limited and skill threshold‐based PCIT, Thomas and Zimmer‐Gembeck (2012) reported that time‐limited PCIT produced equal or superior improvements in child behavior, parent–child communication, and lower child abuse potential scores. Similarly, Jent et al. (2023) found that an 18‐week PCIT session cap was effective in increasing effective parenting skills and reducing children's internalizing and externalizing behaviors. However, a recent meta‐analysis of PCIT with families at high risk for child maltreatment concluded that treatment outcomes were unrelated to the number of sessions completed (Zhang et al., 2024). Here, we attempt to build on previous research using data from a time‐limited RCT of PCIT in child welfare‐involved families by studying the independent and cumulative effects of dosage of each phase of PCIT, that is, CDI phase and PDI phase session dosage on parenting skills and child behavior outcomes.

### The current study

In summary, PCIT focuses on increasing positive, responsive parenting skills and reducing harsh control parenting in CDI sessions, and developing and using safe, effective child management skills in PDI sessions to improve child cooperation and well‐being (Herschell & McNeil, 2005; Skowron & Funderburk, 2022). Meta‐analyses of parenting programs support the inclusion of both phases, showing that improving parent–child relationship quality (e.g., via the CDI‐positive parenting skills) combined with child compliance training and positive discipline practices (e.g., via PDI sessions) is critical for reducing disruptive child behavior (Kaminski & Claussen, 2017; Leijten et al., 2019) and maltreatment risk (Temcheff et al., 2018). However, as limited research to date has examined the PDI phase of treatment, it is essential to understand how both phases of PCIT, the relationship‐enhancing CDI and the positive discipline‐focused PDI, contribute to outcomes for children and caregivers. At the same time, questions remain about the optimal dosage of each phase, especially for families who receive a limited dose of treatment. Therefore, the current study sought to examine the effects of PCIT treatment dosage, specifically CDI phase sessions and PDI phase sessions received, and family outcomes in a child welfare sample. Thus, in the context of an RCT comparing the effectiveness of time‐limited PCIT versus a SAU control condition, we examined the individual and interactive effects of CDI session dosage and PDI session dosage on observed positive and negative parenting skills and child behavioral health outcomes. We hypothesized that the dosage of CDI sessions and PDI sessions families received would each predict gains in parenting skills and child behavior outcomes following treatment, and we made no specific predictions regarding the relative impact of CDI dosage versus PDI session dosage on these outcomes.

## Methods

### Participants

The study sample consisted of *N* = 204 parent–child dyads recruited from the Department of Human Services (DHS) in a midsize city in the U.S. Northwest. At the time of recruitment, all families were involved with DHS child welfare for suspected, substantiated, or risk for child maltreatment. Children were ages 3–7 years at study entry (*M* = 4.76; *SD* = 1.40; one child turned 8 years old several days before their rescheduled pretreatment assessment) and 54.9% boys and 45.1% girls. Most (98%) caregivers were biological parents of their child and primarily (88.2%) mothers. Parents were ages 18–64 (*M* = 32.3; *SD* = 6.4) years old. The majority of sample parents (73.5%) self‐reported experiencing 4+ adverse childhood experiences (ACEs *M* = 5.2, *SD* = 2.7) themselves, and most sample children (69.1%) had experienced 3+ ACEs by study entry. According to the U.S. Department of Health and Human Services 2020 federal poverty guidelines, 78.5% of study families were characterized as living below the poverty line (see Nekkanti et al., 2020 and Skowron et al., 2024 for more information).

### Design and procedure

The study sample was recruited into this RCT of PCIT versus an SAU control condition between April 2016 and June 2019. Prospective participants completed a brief phone screen during which inclusion criteria were assessed (i.e., parent was 18+ years of age and the participating child's biological or custodial caregivers; child was aged 3–7 years at study entry; and parent and child were living together at least 50% of the time and both spoke English). Families headed by any parent with a history of perpetrating child sexual abuse were excluded from the study because PCIT is contraindicated for caregivers who have engaged in sexual abuse. Study procedures were approved by the Institutional Review Board and registered with ClinicalTrials.gov (Skowron et al., 2024). Participation in this study was voluntary.

Caregiver–child dyads completed a pretreatment assessment prior to randomization and a posttreatment assessment ~8 months following study entry (*M* = 7.84; *SD* = 2.34 months), on the same timeline across both conditions. Assessments at each time point included individual and joint measurements of parent and child, video recordings of parent–child interactions during the standard Dyadic Assessment Protocol consisting of a 5‐min child‐led play task, a 5‐min parent‐led play task, and a 5‐min clean‐up task. Recordings were collected, transcribed, and coded using the well‐validated dyadic Parent–Child Interaction Coding System, Fourth Edition (DPICS‐IV, Eyberg, Nelson, Ginn, Bhuiyan, & Boggs, 2013) by trained raters blind to time point of assessment and condition. Parents reported on family demographics, their own and their child's adverse childhood experiences, and perceptions of their child's behavior problems. Families were compensated for attending the assessments and for transportation costs, and provided rest breaks with refreshments and childcare for nonparticipating children (see Nekkanti et al., 2020 for details on study recruitment and procedures).

### Intervention: parent–child interaction therapy

PCIT was delivered in a time‐limited protocol, consistent with other clinical trials in child welfare populations (Chaffin et al., 2004; Thomas & Zimmer‐Gembeck, 2012). All families were offered at least 22 PCIT sessions,^1^ consisting of an intake session, up to 9 CDI sessions (1 skills teaching, 8 coaching), and up to 11 PDI sessions (1 skills teaching, 10 coaching). Session limits were aligned with other published RCTs conducted with child welfare‐involved families (e.g., Chaffin et al., 2004; Thomas & Zimmer‐Gembeck, 2011). Families progressed through treatment (from CDI to PDI; from PDI to graduation) in one of two ways: First, families who achieved parenting skills proficiency could progress to the next phase of treatment on or after the fourth CDI session. Thus, to progress from CDI to PDI, caregivers could demonstrate proficiency in use of PRIDE skills (i.e., producing 10 labeled praises, 10 behavior descriptions, and 10 reflections) in a 5‐min play interaction with their child. To progress from PDI to graduation, caregivers could demonstrate proficiency in use of limit‐setting skills (i.e., during a 5‐min PDI interaction with child, 75% of commands given were effective, with 75% effective follow‐through). Alternatively, families could attend the maximum CDI sessions offered when skills proficiencies were not met, before progressing to the next phase of treatment (PDI). On average, families received a median of 9 CDI sessions (IQR = [5.5, 9]) and 6 PDI sessions (IQR = [0, 9.5]). About 79 families engaged in one or more CDI sessions, and 53 families engaged in at least one or more PDI sessions. PCIT was delivered by eight therapists, including doctoral‐level graduate students, a licensed psychologist and social worker. Therapist training and supervision conformed to PCIT International standards and therapists completed treatment fidelity ratings after each session. Weekly remote consultation and live supervision of therapy sessions was provided by certified trainers at the University of Oklahoma Health Sciences Center. ANOVA tests of therapist effects on pre‐to‐post changes on all coded parenting behaviors were nonsignificant, indicating that any intervention effects on parenting were not attributable to individual differences in therapist effectiveness.

### Services‐as‐usual control condition

The SAU control condition was an ecologically valid, ethical comparison condition in which families accessed typical services provided by DHS child welfare. Thus, families in the SAU control group received a variety of in‐home visitation services, respite childcare, and other individual child and/or adult counseling and support services. Additionally, families had access to other social support services related to housing, food, employment, education, and transportation services. All families, even those in the PCIT condition, retained full access to DHS SAU.

### Measures

#### Observed parenting behaviors

##### Dyadic Parent–Child Interaction Coding System, fourth edition (DPICS‐IV; Eyberg et al., 2013)

Parent and child behaviors during the standard Dyadic Assessment Protocol (i.e., 5‐min each of Child‐Led Play, Parent‐Led Play and a standardized Clean‐Up Task) were video recorded, transcribed, and observationally coded using the well‐validated DPICS‐IV (Nelson & Olsen, 2018). In the context of the Child‐Led Play task, a Positive Parenting score was calculated as the frequency of all parent behaviors that were coded PRIDE skills, namely, Labeled *P*raises (e.g., parent to child, ‘I love how gentle you are being with the blocks’), *R*eflections (e.g., child states, ‘My block is red’; the parent says, ‘Your block is red’), and Behavior *D*escriptions (e.g., while the child builds a road for her cars, the parent may say, ‘You are building a road!’). The Negative Parenting score was calculated as the frequency of all parent behaviors coded as Negative Talk/Criticisms, Questions, and Commands that occurred during the Child‐Led Play task. In the context of the Clean‐Up Task, parents' effective child management skills were assessed via (a) Effective Commands, calculated as the proportion of all parent commands that were ‘good’ compliable direct commands to the child. Children's compliance with parent commands was assessed using (b) Child Compliance, calculated as the proportion of those ‘good’ compliable, direct commands with which the child complied. The Clean‐Up Task (where parents are told to instruct their child to cleanup toys) was chosen as a measure of caregivers' child management skill change because it placed caregivers in a limit‐setting role, which may resemble more closely a natural environment for caregiver–child interactions than Parent‐Led Play (where parents are told to take the lead in deciding what to play with their child). DPICS‐IV coders completed 20 h of intensive training and met regularly to maintain interrater reliability to prevent coder drift. Transcripts and digital video recordings were used to code assessments in Noldus Observer XT. Coders were blinded to condition and assessment timing. Interrater reliability was assessed by independent coding of 20.4% of assessments by two DPICS coders, with 84% agreement at pre‐ and 83% agreement at posttreatment assessments.

#### Child behavior problems

##### Eyberg Child Behavior Inventory (Eyberg & Pincus, 1999)

The 36‐item Eyberg Child Behavior Inventory (ECBI) was used to assess children's disruptive behavior problems. Caregivers reported on the frequency of problem behaviors, using a 7‐point scale to indicate how often the behaviors occur, from (1) never to (7) always (ECBI Intensity score), and whether they considered a behavior to be problematic or not (ECBI Problem score). Raw ECBI scores were standardized, with T‐scores (*M* = 50; *SD* = 10) reported and used for analyses. Higher ECBI scale scores reflect more problems.

In our sample, internal consistency reliability was assessed for the ECBI Intensity and Problem scales at both assessment points. At Time 1, Cronbach's alpha coefficients indicated excellent reliability for both the Intensity scale (α = .941) and the Problem scale (α = .931). Similarly, at Time 3, internal consistency remained high for the Intensity (α = .941) and Problem (α = .925) scales. In addition, test–retest reliability analysis using a two‐way mixed‐effects model with absolute agreement [ICC(3,1); Koo & Li, 2016] yielded intraclass correlation coefficients (ICCs) of .77 for the ECBI Intensity scale and .65 for the ECBI Problem scale, indicating good and moderate stability, respectively for measurements that were ~7.8 months apart (on average; *SD* = 2.3). These results demonstrate strong item homogeneity and reasonable temporal stability of the ECBI scales within this sample.

#### Sociodemographics

At pretreatment, parents provided demographic information about their participating child and themselves (e.g., biological sex, age, ethnicity) and information about their relationship status, highest education completed, household income, and ACEs exposures (Anda, Butchart, Felitti, & Brown, 2010).

#### Parent–child interaction therapy session dosage

CDI dosage was measured by counting the number of CDI sessions families attended. PDI dosage was measured by counting the number of PDI sessions families attended.

### Analytic plan

**Figure 1:**
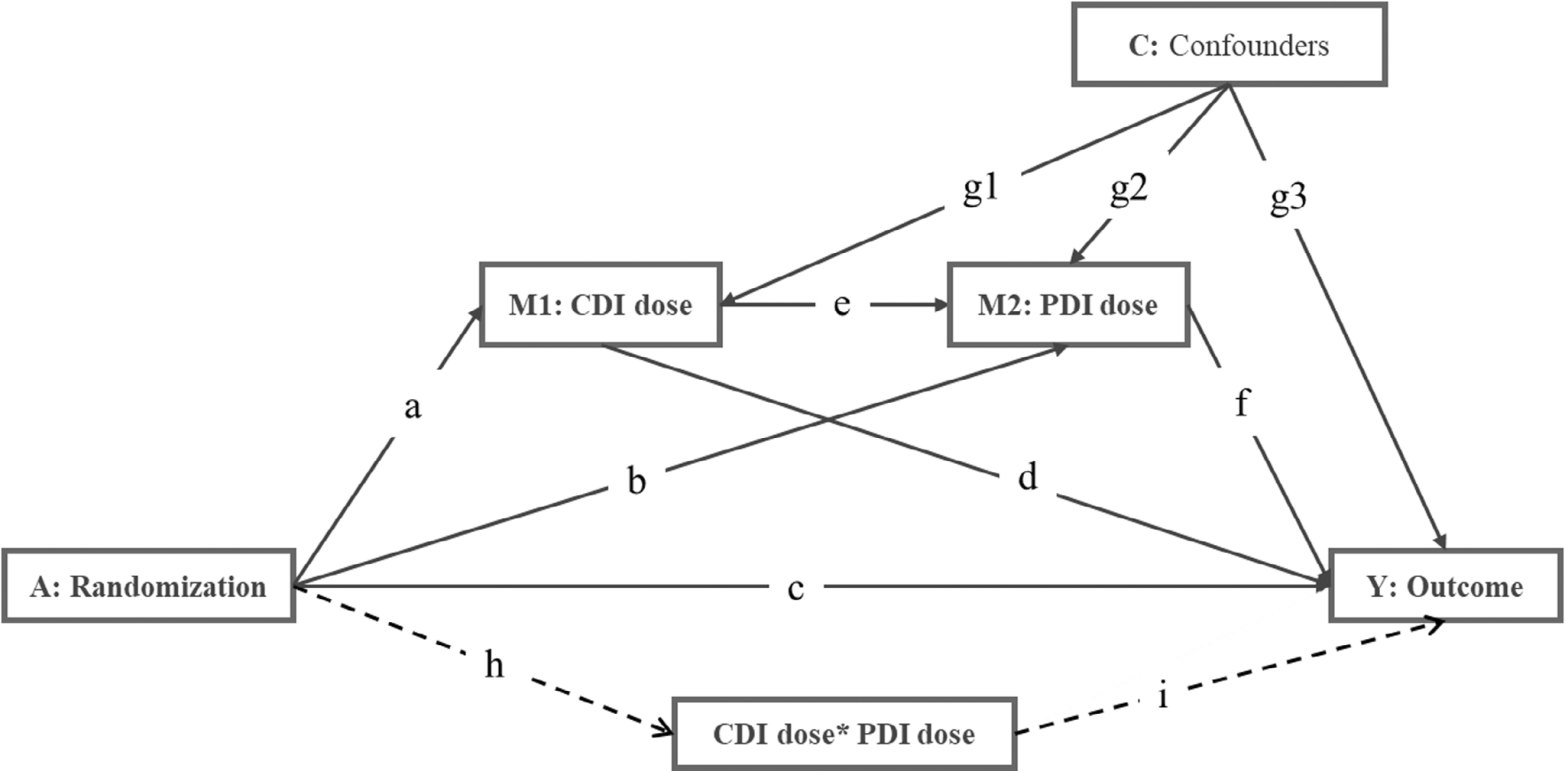
Path diagram of mediation model of treatment dose on outcomes. Path diagram representing the hypothesized sequential mediation modeling structure. A: Randomization, M_1_: first mediator (CDI dose), M_2_: second mediator (PDI dose), Y: outcome, C: confounders of M_1_‐Y, M_2_‐Y, M_1_‐M_2_ associations. The path involving the interaction effect between CDI dose and PDI dose was not included in all models

First, we sought to explore basic CDI phase dose– and PDI phase dose–response relationships within the intervention condition through a series of scatterplots and then conducted multiple linear regression analyses for each outcome variable, with CDI dose and PDI dose as predictors. These exploratory models integrated covariates and considered nonlinear dose–response relationships. Analyses were executed in R using the ‘stats’ package. Second, we used sequential mediation models to test the PCIT intervention's direct effects on parenting and child outcomes, and the indirect effects on outcomes through dosage of CDI phase and PDI phase sessions. As shown in Figure 1, these statistical models tested the following: (1) a direct effect of randomization (intervention vs. control; an intent‐to‐treat effect); (2) an indirect randomization effect, mediated by CDI dosage; (3) sequential mediation through CDI dosage and then PDI dosage; (4) the direct effect from CDI dosage and from PDI dosage to outcomes; and (5) the indirect effect of CDI dosage mediated by PDI dosage on the parenting and child outcomes. PCIT nonengagers were families randomized to the PCIT condition who attended 0 sessions (*n* = 41), and engagers >0 sessions (*n* = 79). For primary analyses, all families were analyzed by randomized condition (PCIT vs. control; *n* = 84 in control), consistent with the intent‐to‐treat principle. Engagement status was examined descriptively only, whereas main intervention effects were analyzed by randomized condition. All models adjusted for the covariates of parent and child sex, child age, and pretreatment scores, which were included to account for any potential confounding effects on CDI dosage, PDI dosage, and outcomes. In addition, we tested an alternative mediation model using the sum of CDI and PDI doses as the outcome variable. This model examined whether the overall number of treatment sessions completed (CDI + PDI combined) accounted for similar mediation effects as the phase‐specific model presented in Figure 1. The findings from this alternative model were compared with the primary model to evaluate whether total treatment dosage provided a more parsimonious explanation of the pathways linking randomization to parenting and child behavior outcomes.

This second set of analyses was executed in Mplus 8.8. All outcome variables and their pretreatment scores were standardized (z‐scores) prior to analysis. Therefore, the unstandardized regression coefficients represent standardized effect sizes (what some call partially standardized/semi standardized coefficients; Cohen, Cohen, West, & Aiken, 2013, pp. 44–46), indicating the change in the outcome (in SD units) per one‐unit change in the predictor (e.g., one session, 1 year, or group difference).

Missing data did not vary by condition on any of the outcome variables tested. Missing variables at pretreatment and posttreatment were imputed using the ‘mice’ package in R (Version 3.3.0; van Buuren & Groothuis‐Oudshoorn, 2011). A predictive mean matching approach was employed for all variables, and the ‘quick_pred’ function within the ‘mice’ package was used to implement a predictor selection strategy. We conducted 40 imputations with 40 iterations each to enhance statistical power and reduce bias, per Graham's (2009) recommendation. Parameters for all analyses were estimated in each of the 40 imputed datasets and pooled using Rubin's rules. All scripts employed in the analyses are accessible upon receipt of a written request.

## Results

### Preliminary analyses

All analyses were conducted with the 204 randomized participants on imputed data (i.e., 40 imputed datasets), including *n* = 120 families randomized to PCIT (79 treatment‐engagers and 41 treatment‐non engagers), and all members of the SAU control group (*n* = 84). Table 1 displays the raw (before imputation) pretreatment and posttreatment means and standard deviations of the outcome variables: DPICS‐coded positive and negative parenting scores during the Child‐Led Play task; DPICS‐coded parent commands and child compliance scores during the Clean‐Up task; and child ECBI behavior intensity and problem scores in both the PCIT and control group families.

**Table 1:** Descriptive statistics of outcomes by group

Outcome	Control (*N* = 84)			PCIT‐not engaged (*N* = 41)			PCIT‐engaged (*N* = 79)		
	*N*	Mean	*SD*	*N*	Mean	*SD*	*N*	Mean	*SD*
Positive parenting behaviors									
Pre	84	2.3	2.3	41	2.2	2.5	79	2.9	3.1
Post	65	1.9	2.1	27	2.3	2.5	64	9.9	9.8
Negative parenting behaviors									
Pre	84	23.6	11.7	41	22.5	11.6	79	24.2	11.9
Post	65	20.8	11.0	27	20.0	10.5	64	12.8	8.9
Child ECBI intensity									
Pre	84	58.6	10.8	40	53.4	8.9	77	57.0	10.2
Post	64	56.8	9.3	26	56.0	8.7	64	50.4	10.1
Child ECBI problems									
Pre	84	58.6	11.8	39	53.2	9.8	76	56.4	11.1
Post	63	55.9	10.8	26	51.9	9.6	62	50.3	9.9
% Good parent commands									
Pre	84	0.48	0.17	41	0.51	0.19	79	0.49	0.16
Post	65	0.51	0.18	27	0.52	0.21	64	0.49	0.17
% Child compliance									
Pre	84	0.70	0.22	41	0.72	0.20	79	0.64	0.28
Post	64	0.76	0.19	27	0.73	0.22	64	0.72	0.27

Positive Parenting and Negative Parenting scores were DPICS‐coded during child‐led play, with higher scores indicating more behaviors. Child ECBI Intensity and number of Problems are standardized *T*‐scores, with higher scores indicating greater intensity and number of problematic child behaviors, respectively. The % Good Parent Commands = proportion of direct, compliable commands of all commands issued and % Child Compliance = proportion of good parent commands complied with, per DPICS coding during clean‐up task. PCIT, parent–child interaction therapy; ECBI, Eyberg child behavior inventory; DPICS, Dyadic Parent–Child Interaction Coding System.

**Figure 2:**
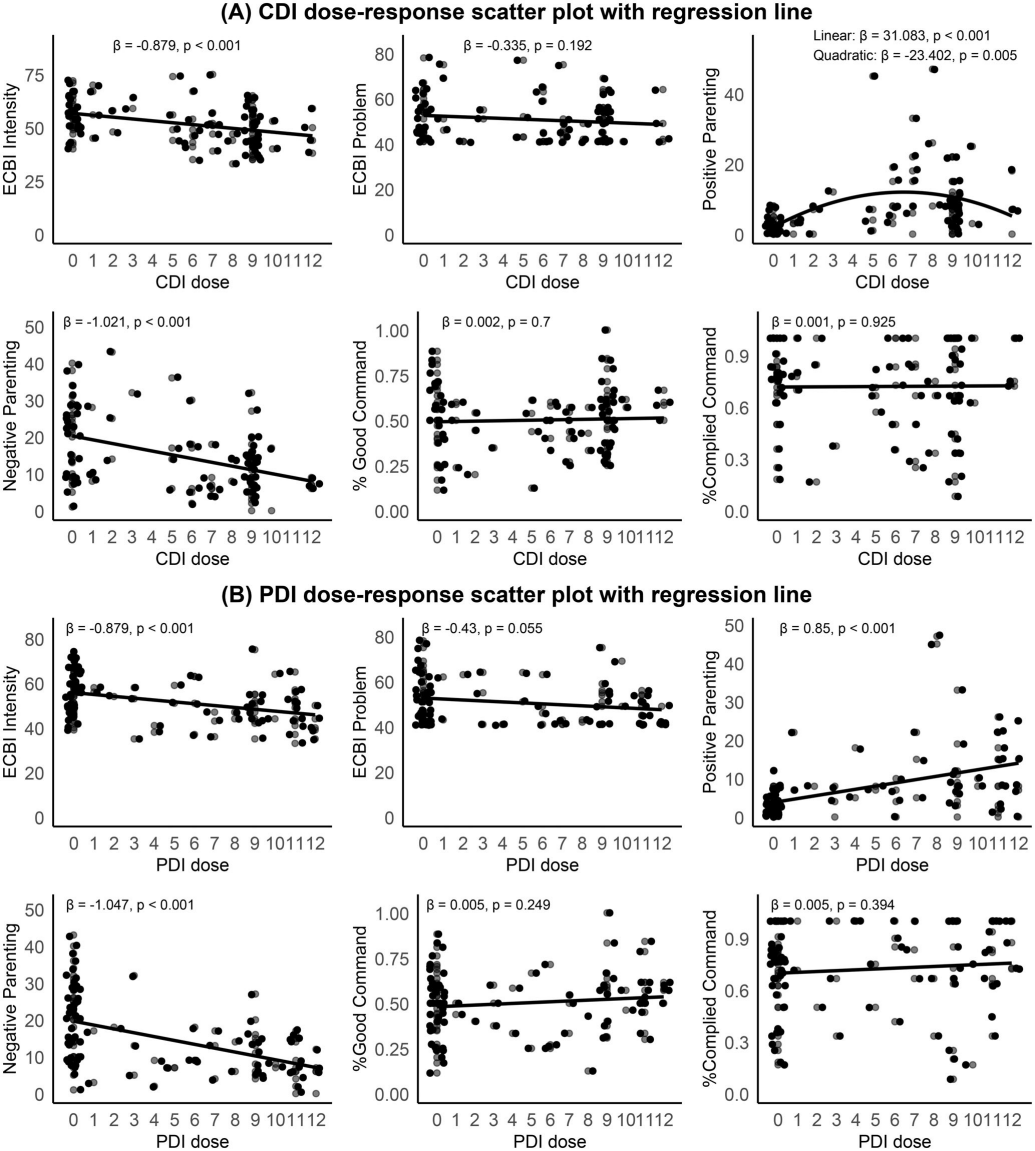
Scatter plots displaying (A) child‐directed interaction (CDI) session dosage and (B) parent‐directed interaction (PDI) session dosage associations with parenting and child outcome variables. *N* = 125 families received 0 CDI sessions

Scatterplots were generated using one of the imputed datasets to visualize the relationships between the dosage of CDI phase sessions and dosage of PDI phase sessions, and each posttreatment outcome variable, providing a complete representation of the data after imputation. As shown in Figure 2A, family participation in (the relationship enhancement‐focused) CDI sessions was associated with more posttreatment Positive Parenting behaviors and fewer Negative Parenting behaviors during Child Led Play, and lower intensity and number of child behavior problems. Figure 2B displays similar associations observed between PDI dosage and outcomes. Specifically, family engagement in (the more positive discipline‐focused) PDI sessions was associated with more posttreatment Positive Parenting and fewer Negative Parenting behaviors during Child‐Led Play, and lower intensity and number of child behavior problems. No discernible associations were observed between CDI dosage or PDI dosage and the percentage of parent Effective Commands or Child Compliance with those commands during Clean Up.

Exploratory multiple regressions were conducted to examine the treatment dose–response relationships, including direct and interactive effects of CDI and PDI dosages on all six outcomes (Table S1). Results did reveal a significant interaction between CDI and PDI sessions on Positive Parenting scores, suggesting the impact of one dose depends on the level of the other for predicting Positive Parenting outcomes. No support was found for the nonlinear CDI and PDI direct effects (after adjusting for the significant interaction term on positive parenting). Based on these findings, we included linear CDI and PDI terms in all subsequent mediation models and for the Positive Parenting outcome included a CDI by PDI interaction term.

### Sequential mediation models

Next, sequential mediation models, as depicted in Figure 1, were conducted to test the mechanisms through which the independent variable (i.e., Randomization to PCIT vs. Control) influences a dependent variable (i.e., outcome) via a chain of two successive mediating variables (i.e., M1: CDI dosage, M2: PDI dosage). In sequential mediation models, the mediators occur in a specific order and thus suggest a causal pathway through which randomization affects the first mediator (CDI dosage; path a), which then affects the second mediator (PDI dosage; path e), which then influences the outcome variable (i.e., parenting and child behavior scores; path f). Parent sex, child sex, and child age were not associated with any mediator or outcome variables but were included in the models along with pretreatment scores as covariates to reduce confounding bias in the focal variable effect estimates.

Results are found in Table 2. Estimates listed in the top right corner are the individual direct effects of the independent variable (Randomization to PCIT or Control; path c), mediators (CDI dose and PDI dose: paths d and f, respectively; and CDI dose * PDI dose interaction: path i), covariates (paths g3), and pretreatment scores on the outcomes. Pretreatment scores were significantly associated with the Negative Parenting scores (β = 0.20, *p* = .02), ECBI Intensity scores (β = 0.52, *p* < .001), ECBI Problem scores (β = 0.42, *p* < .001), and Child Compliance (β = 0.21, *p* = .01), but not with Positive Parenting (β = 0.05, *p* = .52) or Parent Commands (β = 0.13, *p* = .15). No significant direct effects of Randomization were observed on any outcome variables (all *p* > .20). Mediator 1, CDI session dosage, showed a significant direct effect on ECBI Intensity scores (β = −0.07, *p* = .03) and an effect on Positive Parenting scores that was not statistically significant (β = 0.07, *p* = .08). Mediator 2, PDI session dosage, showed significant direct effects on ECBI Intensity scores (β = −.06, *p* = .04), Positive Parenting scores (β = 0.46, *p* = .00), and Negative Parenting scores (β = −0.08, *p* = .01). Further, a significant direct interaction effect between dosage of CDI sessions and dosage of PDI sessions was observed for the Positive Parenting outcome only (β = −0.05, *p* = .01). This interaction complicates the interpretation of the two Positive Parenting direct effects attributed above to CDI session dosage and PDI session dosage. The full effect of PDI dose on Positive Parenting depends on CDI dose (and vice versa) and can be computed using the following formula:

$$\text{Full Effect of}\;\mathrm{PDI}\;\text{dose}=\begin{array}{l} \beta_{\mathrm{PDI}}\times \mathrm{PDI}\;\text{dose}+\beta_{\mathrm{CDI}}\\ \times \mathrm{CDI}\;\text{dose}+\beta_{\mathrm{CDI}\times \mathrm{PDI}}\\ \times \left(\mathrm{CDI}\;\text{dose}\times \mathrm{PDI}\;\text{dose}\right). \end{array}$$

**Table 2:** Direct and indirect effects of randomization and treatment phase dosage on outcomes: results from sequential mediation models

Predictors	Mediators				Outcome variables											
	CDI dose		PDI dose		ECBI intensity		ECBI problem		Positive parenting		Negative parenting		% Good command		% Complied command	
	β	*p*	β	*p*	β	*p*	β	*p*	β	*p*	β	*p*	β	*p*	β	*p*
Randomization	4.74	.00**	−0.09	.73	0.21	.26	−0.04	.86	0.17	.20	−0.02	.92	−0.08	.78	−0.28	.22
CDI dose	–	–	0.79	.00**	−0.07	.03*	−0.02	.64	0.07	.08	−0.02	.57	−0.04	.51	−0.02	.61
PDI dose	–	–	–	–	−0.06	.04*	−0.05	.07	0.46	.00**	−0.08	.01*	0.05	.19	0.05	.18
CDI dose*PDI dose	–	–	–	–	–	–	–	–	−0.05	.01*	–	–	–	–	–	–
Parent sex	0.99	.11	0.33	.51	0.2	.33	0.16	.50	−0.07	.69	−0.07	.77	−0.05	.85	−0.12	.57
Child sex	0.07	.88	0.01	.97	−0.16	.20	−0.05	.70	−0.09	.45	−0.05	.74	0.14	.42	0.2	.21
Child age	−0.13	.43	−0.23	.04*	−0.04	.44	0.02	.70	0.02	.74	0	.95	0.03	.64	−0.01	.85
*Pretreatment score*																
ECBI intensity	0.32	.41	−0.07	.79	0.52	.00**	0.1	.46	0.08	.40	−0.08	.44	−0.13	.30	−0.01	.95
ECBI problem	0.25	.52	0.26	.37	0.08	.46	0.42	.00**	−0.12	.22	0.16	.14	0.04	.78	−0.06	.63
Positive parenting	0.07	.79	−0.32	.13	0.04	.49	0.05	.51	0.05	.52	0.1	.20	0.03	.70	−0.04	.65
Negative parenting	−0.12	.59	0.13	.45	0.05	.49	0.09	.23	0.15	.04*	0.2	.02*	−0.16	.07	0	.98
% Good command	0.27	.28	−0.07	.68	0.08	.18	0.1	.14	−0.06	.26	−0.11	.17	0.13	.15	−0.05	.55
% Complied command	−0.22	.35	0.23	.21	−0.01	.92	−0.07	.28	0.05	.54	−0.08	.25	−0.05	.50	0.21	.01*
CDI dose*PDI dose																
Randomization	–	–	–	–	–	–	–	–	30.86	.00**	–	–	–	–	–	–
*Direct and indirect impacts of randomization on outcome*																
Total					−0.32	.02*	−0.31	.04*	0.78	.00**	−0.4	.01*	−0.06	.72	−0.2	.23
	Direct				0.21	.26	−0.04	.86	0.17	.20	−0.02	.92	−0.08	.78	−0.28	.22
	Total indirect				−0.53	.00**	−0.28	.02*	0.61	.00**	−0.38	.00**	0.01	.92	0.08	.58
		Randomization → CDI dose → Outcome			−0.33	.04*	−0.08	.64	0.33	.09	−0.1	.57	−0.16	.51	−0.11	.61
		Randomization → PDI dose → Outcome			0	.76	0	.76	−0.04	.74	0.01	.74	0	.78	0	.79
		Randomization → CDI → PDI → Outcome			−0.21	.05*	−0.2	.08	1.74	.01*	−0.29	.01*	0.18	.20	0.19	.20
		Randomization → CDI*PDI → Outcome			–	–	–	–	−1.42	.01*	–	–	–	–	–	–
Direct and indirect effects of treatment dosage on outcome																
Total					−0.11	.00**	−0.06	.01*	0.44	.00**	−0.08	.00**	0	.90	0.02	.55
	Direct				−0.07	.03*	−0.02	.64	0.07	.08	−0.02	.57	−0.04	.51	−0.02	.61
	Total indirect				−0.04	.05*	−0.04	.07	0.37	.00**	−0.06	.01*	0.04	.20	0.04	.20
		CDI dose → PDI dose → Outcome			−0.04	.05*	−0.04	.07	0.37	.00**	−0.06	.01*	0.04	.20	0.04	.20

(1) * <.05; ** <.01; (2) The CDI dose*PDI dose interaction effect on Positive Parenting is shown here. (3) βs are unstandardized coefficients. The outcomes and pretreatment scores were standardized before being entered into the model; therefore, the unstandardized coefficients can be interpreted as effect sizes (i.e., the expected change in the outcome in standard deviation units per one‐unit change in the predictor).

**Figure 3:**
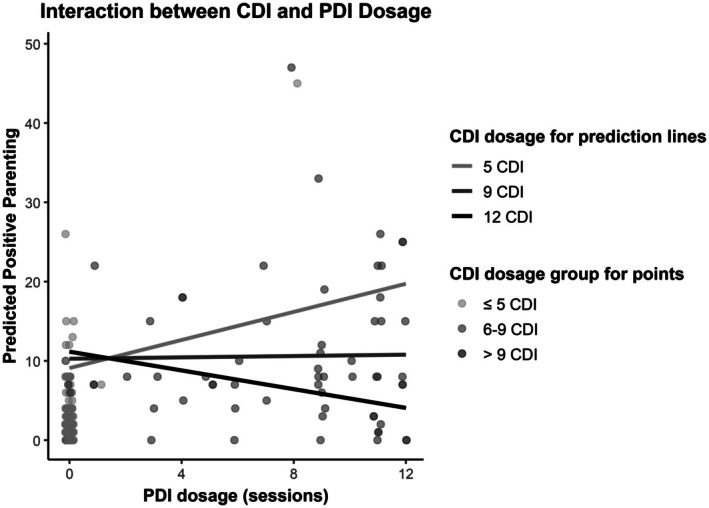
Interaction of CDI and PDI dosage predicting Positive Parenting score. Predicted Positive Parenting scores across PDI dosage, plotted separately for CDI dosage levels (5, 9, and 12 sessions). Points represent observed values grouped by CDI dosage categories (≤5, 6–9, >9 sessions) in the raw dataset

Figure 3 illustrates how the full effect of PDI dose on posttreatment Positive Parenting scores changes across three different values of CDI dose. For ease of interpretation, we plotted predicted slopes at three representative CDI values (5, 9, and 12 sessions), chosen to reflect lower, average, and higher CDI dosage levels observed in the sample. To visualize the observed data alongside the model‐based estimates, participants were grouped into three CDI categories (≤5, 6–9, and >9 sessions) and plotted as points with different shading. As shown, when the CDI session total equals 5, the statistical models predict an increase in the Positive Parenting score with each additional PDI session. However, when the CDI sessions total 9, the predicted Positive Parenting score remains stable regardless of the PDI dosage received. As the CDI total surpasses 9, Positive Parenting score predictions actually decline with each additional PDI session (e.g., notice the negative slope for the CDI = 12 prediction line).

Turning to the prediction of the CDI mediator (top left panel of Table 2), results show that only Randomization was a significant predictor of variation in the number of CDI sessions completed (β = 4.74, *p* < .001). No covariates or pretreatment outcome scores significantly predicted the CDI dose mediator. In terms of the PDI mediator, Randomization did not directly predict the number of PDI sessions completed (β = −0.09, *p* = .73, ns); however, the count of CDI sessions received was a significant predictor of PDI dose (β = 0.79, *p* < .001), where higher CDI session counts were associated with greater PDI sessions completed. Child age also significantly predicted PDI dose (β = −0.23, *p* = .04), indicating families with older children participated in fewer PDI sessions. No other covariates or pretreatment scores predicted PDI dosage.

### Dosage of CDI and PDI sessions mediates intervention effects on outcomes

As given in Table 2 (1st row), there were no direct effects of randomization on the treatment or control conditions for any of the outcomes. However, the total effects of randomization (see Table 2, ‘Total and Indirect Effects of Randomization on Outcome’), including direct and all dosage‐mediated indirect effects, indicate the PCIT group achieved significantly greater Positive Parenting skills (β = 0.78, *p* < .001), lower Negative Parenting behaviors (β = −0.40, *p* < .01), and reductions in child behavior problems per ECBI Intensity scores (β = −0.32, *p* = .02) and ECBI Problem scores (β = −0.31, *p* = .04) at post intervention. Given the small (nonsignificant) direct effects of Randomization on all outcomes, these results support the presumption that the effect of PCIT on outcomes largely depends on (are fully mediated by) the completion of PDI and/or CDI sessions. The Table 2 row labeled ‘Randomization → CDI dose → PDI dose → Outcome’ displays results of the sequential indirect effect of the PCIT intervention through dosage of CDI sessions and subsequent PDI sessions (Figure 1 paths a–e–f). This pathway was statistically significant in predicting greater positive parenting skills (Indirect effect: β = 1.74, *p* < 0.01), reductions in negative parenting (Indirect effect: β = −0.29, *p* < .01), and reductions in child behavior problems per ECBI Intensity scores (Indirect effect: β = −0.21, *p* < .05) at posttreatment. This same pathway predicted a nonsignificant reduction in ECBI Problem scores (Indirect effect: β = −0.20, *p* = .08) at posttreatment. PCIT Randomization also exerted a significant indirect effect on reductions in ECBI Intensity scores through the mediating CDI dosage pathway alone (β = −0.33, *p* < .05). No other indirect effects of Randomization through the CDI dose or PDI dose pathways alone were significant predictors of the outcomes (i.e., all mediation paths predicting quality of parenting commands and rates of child compliance were nonsignificant). Finally, it is worth noting the full impact of PCIT Randomization on positive parenting skills was dependent on the significant interaction between CDI dose and PDI dose mentioned earlier. Randomization‐induced variability in CDI dose and PDI dose led to nonadditive influences on positive parenting skills, and thus, the total indirect effect of Randomization through CDI dosage depended on the amount of PDI sessions (see Figure 1, paths h–i). This is perhaps best understood with the aid of a heatmap (see Figure S1), which shows that maximal Positive Parenting predictions correspond to high counts of PDI sessions and low counts of CDI sessions whereas minimal predictions involve a large number of both PDI and CDI sessions. Importantly, this maximal prediction space falls ‘out‐of‐sample,’ (i.e., no participants manifested this combination of low CDI and high PDI session counts; see Figure S2). If we restrict the prediction space to in‐sample observations (see Figure S3, ‘Positive Parenting’ panel), we notice the optimal combinations involved a medium‐high number of CDI sessions (≈6) and a maximum number of PDI sessions (12), with low numbers of either phase or a high‐high combination leading to suboptimal improvements.

### Alternative mediation models

The sequential mediation model (Table 2) examined the indirect effects of randomization on outcomes through CDI dose, PDI dose, and their interaction (CDI × PDI), whereas the alternative model tested total PCIT dose (CDI + PDI combined) as a single mediator (Table S2). Across both models, randomization did not have significant direct effects on parenting or child outcomes, but indirect effects through treatment dose were significant and consistent across domains. The alternative total‐dose model showed significant indirect effects for ECBI Intensity (β = −0.52, *p* < .001), ECBI Problem (β = −0.30, *p* < .001), Positive Parenting (β = 0.51, *p* < .001), and Negative Parenting (β = −0.42, *p* < .001), closely mirroring results from the sequential model.

The sequential model, however, provided greater specificity, showing that CDI dose played a stronger mediating role than PDI dose, and that their interaction (CDI × PDI) significantly predicted Positive Parenting (β = 30.86, *p* < .001). Taken together, both models indicate that greater PCIT exposure mediated improvements in parenting and child behavior, but the sequential model was retained as the primary analytic model for its clearer alignment with the theoretical structure of PCIT and its ability to distinguish phase‐specific pathways.

## Discussion

PCIT is a multifaceted therapeutic intervention designed to improve parent–child interactions and reduce child behavior problems. Understanding dose–response effects is critical for optimizing intervention delivery and gaining insight into the underlying mechanisms of this therapy. Therefore, in our comparison of the effects of PCIT versus an SAU control in a child welfare sample, we examined how PCIT session dosage impacted parenting quality and children's behavioral health outcomes. Specifically, we tested the independent and joint impacts of session dosage in each of PCIT's two phases, CDI (a phase that is focused on building a warm relationship between caregiver and child) and PDI (a phase that is focused on honing caregivers' safe, effective discipline skills), on parent and child outcomes. Overall, we found that PCIT, indirectly through participation in more of the relationship building CDI sessions followed by engagement in more positive discipline PDI sessions, had significant effects on increasing positive parenting behavior, decreasing negative parenting behavior, and reducing both child behavior problems and how serious caregivers perceived these problems to be. For positive parenting outcomes specifically, we also found an interaction between doses of each phase of PCIT, such that the number of warm relationship‐building (CDI) sessions a family received and the number of positive discipline (PDI) sessions a family received not only built upon each other but also uniquely impacted positive parenting through their interaction. These findings underscore the importance of the PDI phase of the intervention in the overall effectiveness of PCIT with child welfare‐involved families.

### Parenting skill outcomes

We first examined the impact of CDI dose, the warm relationship‐building phase sessions, and PDI dose, the safe discipline skills phase sessions, on parenting skills at posttreatment. We found that session dosage for each phase of treatment predicted gains in negative and positive parenting skills. Multiple regression analyses showed that reductions in negative parenting behaviors at posttreatment were predicted by greater PDI dose, and not *uniquely* by CDI dose. Families who engaged in more PDI sessions saw decreases in negative parenting behaviors, suggesting that this safe discipline‐focused phase may be of particular importance for addressing negative parenting. It is important to note, however, that CDI sessions in this study always preceded the PDI phase, and the CDI session count strongly predicted the PDI session count. While CDI did not uniquely predict negative parenting, its indirect effect through PDI was significant (Figure 1, path e–f; β = −0.06; *p* < 0.01). Due to this temporal dependency, it is difficult to separate out the relative importance of each session type.

For positive parenting skills, we saw a significant interaction between CDI and PDI sessions, suggesting the impact of one phase's dose on these skills depended on the dose of the other phase. Specifically for this sample of child welfare‐involved families, as the number of CDI sessions increased, the impact of PDI sessions on positive parenting skills declined, having virtually no influence once the CDI session count reached 9 and even some minor detriment once the count reached 12. These findings suggest that those who received an intermediate dose of CDI benefited more from a greater number of PDI sessions, while for families who participated in the most CDI sessions, additional PDI sessions either did not benefit families or paradoxically hindered gains in positive parenting skills.^2^

There are several possible explanations for these positive parenting skills findings. First, findings are consistent with previous studies suggesting that brief interventions may be of particular benefit to high‐risk families (e.g., Bakermans‐Kranenburg et al., 2003; Thomas & Zimmer‐Gembeck, 2012). It also is possible that ceiling effects may be present (i.e., more CDI sessions are not necessarily better for family outcomes). However, there are likely additional family‐level factors that require careful consideration. Families progressed to PDI phase sessions when either (a) CDI parenting skill thresholds were achieved or (b) the maximum number of allowed CDI sessions was completed. As such, those who remained in the CDI phase through the maximum allotment were also more likely to be those who had not yet achieved positive parenting skills benchmarks. It is possible that family‐level factor differences moderate the dosage of CDI needed to reach skill benchmarks. Further research is required to determine whether a dosage inflection point exists. In other words, is there a number of sessions at which CDI sessions no longer effectively improve PRIDE skills and a transition to PDI is prudent? If so, does participation in additional sessions above and beyond that inflection point offer diminishing returns for family outcomes? Further research is needed to identify salient individual differences that may influence the impact of session dosage (e.g., Zhang et al., 2024) on family progress through both phases of PCIT treatment, access to sufficient PDI phase sessions, and optimize families' posttreatment outcomes.

Although randomization itself was not a significant predictor of outcomes in this study, assignment to PCIT vs. the control condition did significantly predict outcomes through the series of intermediate dosage variables (CDI dose followed by PDI dose). Specifically, PCIT randomization significantly impacted positive and negative parenting skills at outcome through these indirect, dosage pathways. While, by itself, CDI dose did not exert a direct impact on positive and negative parenting outcomes, a noteworthy indirect effect of CDI dose on outcomes was observed through the dosage of PDI sessions families completed, suggesting that the number of CDI sessions completed influences the outcome indirectly through the number of PDI sessions completed, highlighting the interconnected roles of both mediators in shaping parenting skills and child behavior outcomes. These findings provide new evidence to suggest that the benefits of PCIT for child welfare‐involved families may be due to influences from both the warm relationship‐building CDI phase and the positive discipline, limit setting‐focused PDI phase. To date, most research on PCIT components has focused on the impact of CDI sessions (e.g., Barnett et al., 2015; Hakman et al., 2009; Lieneman et al., 2019), with notably less literature available regarding the role of limit‐setting PDI sessions on child and caregiver outcomes. The results of this study highlight the importance of these positive discipline skills‐focused sessions and provide new evidence to suggest that the PDI phase of treatment may play a crucial role in improving caregiver outcomes in child welfare‐involved families.

### Child behavior outcomes

We also assessed the impact of CDI and PDI sessions on child behavior at posttreatment and found that families who engaged in more of either phase of the intervention reported decreases in child behavior problems. Child problem behavior was measured in two ways: frequency of the behavior (via ECBI Intensity score) and how problematic caregivers view the behavior to be (via ECBI Problem score). The intervention reduced both the frequency of problem child behavior and how problematic caregivers reported that behavior to be, among families who participated in more CDI‐positive parenting and PDI limit setting sessions. In other words, the more sessions a family completed of both the warm relationship‐enhancing CDI phase and the positive discipline skill‐building PDI phase, the less frequently child behavior problems occurred and the less troublesome caregivers felt those problems were on outcome. Thus, it appears that in the context of time‐limited PCIT, dosage mattered: more sessions were better for child behavior outcomes than fewer sessions, and both CDI and PDI sessions impacted this particular outcome. Here, we see further evidence that having exposure to the PDI phase of treatment seems to matter for reducing the frequency of child behavior problems (cf. Eisenstadt et al., 1993). Perhaps PDI's emphasis on positive discipline skills is critical for increasing caregivers' efficacy in limit‐setting and effectively responding to their child's misbehavior, leading to this observed decrease in problem child behavior.

We also saw evidence that PCIT had a significant impact on lowering the frequency of child behavior problems when mediated only by the number of warm, relationship‐building CDI sessions that families received. Not only is dosage relevant to improving these particular outcomes, but CDI seems to be particularly important for lowering the intensity of child behavior problems. CDI emphasizes positive parenting skills and the planned ignoring of minor misbehavior, allowing parents to focus attention on more desirable child behaviors (Eyberg, Nelson, & Boggs, 2008; Skowron & Funderburk, 2022). This particular emphasis appears to be having its intended effect: the more CDI sessions received, and therefore the more opportunities a caregiver had to practice removing attention from problem behavior and focusing on desirable behavior instead, the fewer child problem behaviors were reported. In sum, both CDI and PDI dosage acted as crucial mediators between randomization and child behavior outcomes: these findings underscore the importance of structured intervention doses in achieving positive results in child behavior and parenting practices, highlighting the need for tailored, phase‐specific dosing to optimize therapeutic outcomes.

### Command and compliance outcomes

Across all analyses, we found no impact of CDI or PDI session dosage on the quality or quantity of commands given by parents during the clean‐up task, nor on the rates of child compliance to parent commands. These findings ran counter to our predictions but were not entirely surprising. Therefore, capturing change in the number of commands given may not be indicative of progress for some families, including child welfare‐involved families where an overreliance on harsh control parenting exists. Use of developmentally appropriate commands helps caregivers set realistic behavioral expectations for their children (Eyberg et al., 2008); however, some new evidence suggests that child welfare‐involved caregivers participating in PCIT who use more warm, responsive parenting behaviors during limit‐setting also achieve greater child compliance when using fewer commands (Rosen et al., 2026). Although outside the scope of the current study, future research on parent command–child compliance interactions should consider examining the rate of follow‐through (i.e., caregivers correctly responding to a child's compliance by offering specific praise, or their noncompliance by initiating the time‐out sequence), as this may be a more precise measure of caregiver skill growth than the rate of commands. Further, the DPICS coding system captures child compliance immediately following a caregiver command but does not include other dimensions of child cooperation. For example, consider a child who immediately complies with a caregiver's instruction (‘Put the crayons in the box, please.’). Afterwards, without any additional commands given, the child spontaneously cleans up the blocks and the Lincoln Logs, and the caregiver reinforces this cooperation with warmth and responsiveness. This proactive, prosocial initiative by the child and effective positive reinforcement by the caregiver are not currently captured in command–compliance interactions as defined by the DPICS coding system. It may not be sufficient to consider only the quantity of effective commands and the rate of child compliance immediately following a command. Future research can broaden the lens by which command–compliance interactions are viewed so as not to miss other important collaborative exchanges between caregiver and child: possibly PCIT dosage plays a role in influencing other dimensions of parent–child cooperation.

### Limitations

There are several limitations in this work that warrant attention. First, as with all causal inference, the intent‐to‐treat (ITT) approach analyzed participants' data based on their initial assignment group, regardless of level of adherence or, in other words, whether they ultimately received the assigned intervention or not. Causal inference in ITT analysis relies on the consistency principle, a fundamental concept in causal inference, that holds that the effect of a treatment on an outcome should be consistent across different levels of adherence to the treatments to make valid causal claims about the effects of treatments on outcomes. However, our sequential mediation model does not address potential selection bias stemming from the fact that some families randomized to the intervention did not participate in the treatment. Thus, these results have built‐in selection effects that impact the findings. Furthermore, it was not possible to randomize families to varying dosages of CDI or PDI sessions. Finally, given that PCIT is a manualized intervention outlined by PCIT International Guidelines, experience in CDI sessions was a precondition for receiving subsequent PDI sessions (Eyberg & Funderburk, 2011).

Second, there are several limitations associated with the multiple linear regressions conducted to explore the direct CDI dose‐ and PDI dose–response relationships with the outcomes. These analyses neither modeled the randomization effect nor sequential delivery of CDI sessions that (always) preceded PDI sessions per standard PCIT intervention delivery. We therefore incorporated a sequential mediation model to address these limitations by delving into the sequential pathways through which an initial variable (the independent variable randomization) influences an outcome variable via a series of intermediate variables (the mediators CDI dosage and PDI dosage). This approach allows for a more comprehensive understanding of the complex interplay between randomization to condition, PCIT treatment dosage (where CDI and PDI operate sequentially with PDI dose enhancing the effects of CDI dose), and outcomes. By embracing such a model, we can better assess the cumulative impact of these mediators on the outcome variable, thereby enhancing the validity and depth of our analysis.

Third, answering questions about CDI and PDI dosage broadly is complicated for a variety of reasons. The amount of treatment offered to families varies significantly across studies, making it challenging to generalize findings across different contexts. In community clinics, as per PCIT protocol, it is standard to proceed to the PDI phase only after parents have achieved parenting skill benchmarks in the positive parenting skills coached in CDI sessions (Hembree‐Kigin & McNeil, 1995). Therefore, in these community studies, many families have received far greater than the average number of CDI sessions before moving to PDI. However, RCTs tend to establish standardized treatment in terms of dosage and typically cap each phase at a specific number of sessions (e.g., Chaffin et al., 2004, 2011; Skowron et al., 2024; Thomas & Zimmer‐Gembeck, 2012). Phase length varies across studies by design, which presents additional challenges to the interpretation of each phase's effects on outcomes and thus efforts to generalize findings.

Though it was beyond the scope of the current study to examine whether session‐by‐session change in observed parenting skills (e.g., PRIDE skills, rate of effective commands) over time would predict incremental gains in child behavior outcomes (e.g., ECBI scores, child compliance), future research should consider how dosage might play a role in the impact of caregiver skill development and their trajectories of change on child behavior outcomes. Finally, the current study used session limits and parenting skill benchmarks to transition families through treatment. While parenting skill benchmarks are widely implemented in clinical settings, these may not be strongly substantiated by current large‐scale RCTs. Future work should examine the role of PCIT phase transition criteria in improving outcomes for caregivers and children.

## Conclusion

While the benefits of PCIT are well documented, treatment attrition remains a key concern when working with child welfare‐involved families (Batzer et al., 2018; Chaffin et al., 2009; Skoranski et al., 2021). Dropout risk is associated with lower socioeconomic status (Fernandez & Eyberg, 2009; Kazdin, Mazurick, & Bass, 1993; McCabe, 2002) and higher rates of negative parenting at pretreatment (Fernandez & Eyberg, 2009; Skoranski et al., 2021; Werba, Eyberg, Boggs, & Algina, 2006), among other factors. However, attrition in PCIT may also be influenced by the structure of the intervention itself. Because families do not typically advance to the PDI phase until they reach CDI parenting skill benchmarks, some may become discouraged by a lengthy CDI phase and discontinue treatment before receiving PDI. As a result, these families may miss out on critical training in positive discipline strategies, training that is theorized to be of particular clinical significance for reducing risk for child maltreatment by providing child welfare‐involved parents with safe and effective limit‐setting tools and strategies for addressing disruptive child behavior problems (Eyberg et al., 2001; Herschell & McNeil, 2005; Skowron & Funderburk, 2022). Our findings – that CDI contributes to gains in positive parenting and reductions in negative parenting and child behavior problems through participation in PDI – underscore the distinct importance of the PDI phase for this population. Given these findings and evidence that behavioral parenting interventions with a modest number of sessions may be preferable for improving outcomes in high‐risk families (Bakermans‐Kranenburg et al., 2003), it may be worth considering whether modifications to PCIT's structure could help ensure more families access this later‐phase content. For example, in the context of time‐limited PCIT, a structured session cap on CDI could provide a pathway for more families to benefit from PDI without requiring them to meet parenting skill benchmarks before progressing, allowing them access to this crucial phase of treatment (Ramos et al., 2025; Thomas & Zimmer‐Gembeck, 2012). At the very least, findings like these could help set realistic expectations for child welfare‐involved families engaging in PCIT by emphasizing the benefits of the PDI phase, potentially improving retention rates within this population.

As the CDI phase of PCIT focuses on increasing parental warmth and strengthening the parent–child relationship, and the PDI phase emphasizes limit‐setting and practicing safe and effective discipline techniques (Skowron & Funderburk, 2022), it has been theorized that these phases impact outcomes in unique ways. The results of this study provide evidence to support this theory. Specifically, engagement in CDI and PDI sessions appears to reduce children's behavioral problems, enhance positive parenting, and decrease negative parenting behaviors. In the context of a time‐limited treatment, more sessions devoted to increasing warm parenting behavior equips parents with the resources to strengthen the relationship with their child, and more sessions devoted to positive discipline skills practice foster positive parenting and reduce the need for negative parenting practices. Together, the combined impact of the CDI and PDI phases is essential for promoting positive outcomes in both parents and children, with PDI playing an important, unique role in bolstering the gains made during CDI and ensuring improvements in parenting practices and child behavior. This study provides an important first step in understanding the optimal dosage of PCIT needed to maximize benefits, preventing over‐ or under‐treatment and supporting efficient resource allocation. While further research is needed to fully explore and confirm these effects, our findings lay a valuable foundation for understanding the mechanisms of PCIT and ensuring it best serves child welfare‐involved families.

## Ethical considerations

Informed consent has been appropriately obtained. The protocols were approved by the Institutional Review Boards of the University of Oregon (IRB #07102014) and the Oregon Department of Human Services (#188). Date of IRB approval: February 17, 2016.

## Trial registration

The clinicaltrials.gov reference number for the CAPS study is NCT02684903. The date of approval for the ClinicalTrials.gov registration is February 17, 2016.

**Key points**

**What's known?**

Parent–Child Interaction Therapy (PCIT) is an evidence‐based parenting intervention that has been effectively implemented in child welfare‐involved families to strengthen positive parenting and reduce child maltreatment risk and recidivism rates. PCIT has two phases: Child‐directed interaction (CDI), which focuses on strengthening a warm relationship between parent and child, and parent‐directed interaction (PDI), which focuses on teaching safe, effective discipline skills.

**What's new?**

Less is known about phase‐specific effects of PCIT on parent and child outcomes. Results from our sequential mediation models show that the PCIT intervention exerted significant indirect effects on increased positive parenting skills and decreased negative parenting behaviors and child behavior problems through higher dosage of relationship‐enhancing CDI sessions followed by higher dosage of safe discipline‐focused PDI sessions. Further, CDI dosage interacted with PDI dosage to predict greater gains in positive parenting skills outcomes.

**What's relevant?**

These results contribute new insights into the pathways through which PCIT shapes outcomes in a sample of child welfare‐involved families. Findings also highlight the significant unique contribution that limit‐setting‐oriented PDI, a relatively understudied phase of PCIT, plays in enhancing positive parenting skills and mitigating child behavior problems.

## Supporting information


**Table S1.** Dose–response relationship: results from multiple linear regression.
**Table S2.** Results from the alternative mediation model with PCIT dose defined as CDI dose + PDI dose.
**Figure S1.** Impact of CDI and PDI dosage on predicted changes in positive parenting scores in theoretical scenarios.
**Figure S2.** Count of PCIT‐assigned children in scenarios receiving various combinations of CDI and PDI dosages in the actual data.
**Figure S3.** Impact of CDI and PDI dosage on predicted change in outcomes in the actual data.

## Data Availability

Data for this manuscript were drawn from an NIH‐funded randomized clinical trial of PCIT with child welfare families, which was pre‐registered at ClinicalTrials.gov. The study protocol was reported in Nekkanti et al. (2020), and primary outcomes addressing the study's central aims were reported in the article by Skowron et al. (2024) and Lyons, Nekkanti, Funderburk, and Skowron (2022). Articles reporting findings based on pre‐intervention (T1) measures include Skoranski et al. (2021) and Zhang et al. (2024). The current article represents a follow‐up study about the effects of treatment dosage, specifically variation in the number of sessions families completed in each phase of PCIT (i.e., child‐directed interaction session dose and parent‐directed, positive discipline session dose) on parenting skills and child behavior outcomes.

## Endnotes

1 Of note, four intervention condition families received greater than the 22 total sessions (i.e., at *n* = 23 to 30 sessions): These families were among the earliest randomized to the intervention (for more information on the study protocol, see Nekkanti et al., 2020).

2 While families who received maximum amounts of CDI sessions did not report large gains in positive parenting skills, they did achieve significant reductions in negative, aversive parenting and disruptive child behavior problems.
