# Supplementary material for: Effects of parent–child interaction therapy dosage on child and parent outcomes: differentiating child‐directed interaction and parent‐directed interaction session impacts in child welfare‐involved families
Source: J Child Psychol Psychiatry. 2026 Feb 3;67(8):1224–43. doi: 10.1111/jcpp.70106 (PMC13341367; doi:10.1111/jcpp.70106)

**Effects of Parent Child Interaction Therapy dosage on child and parent outcomes: Differentiating Child-Directed Interaction and Parent-Directed Interaction session impacts in child welfare-involved families**

**Supporting Information**

Table S1. Dose-Response Relationship: Results from Multiple Linear Regression.

|  | **ECBI  Intensity** | | | **ECBI  Problem** | | | **Positive  Parenting** | | | **Negative  Parenting** | | | **%Good  Commands** | | | **%Complied  Commands** | | | |
| --- | --- | --- | --- | --- | --- | --- | --- | --- | --- | --- | --- | --- | --- | --- | --- | --- | --- | --- | --- |
| **Predictors** | **β** | **95% CI** | ***p*** | **β** | **95% CI** | ***p*** | **β** | **95% CI** | ***p*** | **β** | **95% CI** | ***p*** | **β** | **95% CI** | ***p*** | **β** | **95% CI** | ***p*** |  |
| CDI dose | -0.05 | [-0.11,0.00] | *.06* | -0.02 | [-0.08,0.04] | *.50* | 0.08 | [0.01,0.16] | *.02** | -0.02 | [-0.09,0.04] | *.47* | -0.04 | [-0.12,0.04] | *.31* | -0.04 | [-0.12,0.03] | *.26* |  |
| PDI dose | -0.06 | [-0.11, -0.00] | *.04** | -0.05 | [-0.11,0.00] | *.06* | 0.47 | [0.19,0.76] | *.00** | -0.08 | [-0.13,-0.02] | *.01** | 0.05 | [-0.02,0.12] | *.17* | 0.05 | [-0.02,0.12] | *.16* |  |
| CDI dose*PDI dose |  |  |  |  |  |  | -0.05 | [-0.08, -0.02] | *.00** |  |  |  |  |  |  |  |  |  |  |
| Parent sex | 0.19 | [-0.19,0.57] | *.32* | 0.16 | [-0.29,0.61] | *.48* | -0.07 | [-0.40,0.25] | *.66* | -0.07 | [-0.54,0.40] | *.76* | -0.04 | [-0.52,0.43] | *.85* | -0.12 | [-0.53,0.30] | *.59* |  |
| Child sex | -0.15 | [-0.39,0.09] | *.21* | -0.06 | [-0.32,0.21] | *.69* | -0.08 | [-0.31,0.14] | *.48* | -0.05 | [-0.33,0.23] | *.73* | 0.13 | [-0.19,0.45] | *.41* | 0.19 | [-0.12,0.50] | *.22* |  |
| Child age | -0.04 | [-0.14,0.06] | *.44* | 0.02 | [-0.09,0.13] | *.70* | 0.02 | [-0.10,0.14] | *.73* | 0.00 | [-0.11,0.12] | *.95* | 0.03 | [-0.09,0.15] | *.63* | -0.01 | [-0.13,0.11] | *.84* |  |
| *Pretreatment score* |  |  |  |  |  |  |  |  |  |  |  |  |  |  |  |  |  |  |  |
| ECBI Intensity | 0.51 | [0.32,0.71] | *.00** | 0.10 | [-0.15,0.35] | *.43* | 0.07 | [-0.10,0.25] | *.41* | -0.08 | [-0.28,0.12] | *.43* | -0.12 | [-0.36,0.11] | *.31* | 0.00 | [-0.23,0.24] | *.98* |  |
| ECBI Problem | 0.07 | [-0.14,0.29] | *.51* | 0.42 | [0.17,0.67] | *.00** | -0.13 | [-0.32,0.06] | *.18* | 0.16 | [-0.04,0.37] | *.12* | 0.04 | [-0.20,0.28] | *.75* | -0.05 | [-0.29,0.19] | *.70* |  |
| Positive Parenting | 0.04 | [-0.07,0.16] | *.44* | 0.05 | [-0.09,0.19] | *.49* | 0.06 | [-0.10,0.21] | *.49* | 0.10 | [-0.05,0.25] | *.18* | 0.03 | [-0.13,0.19] | *.69* | -0.04 | [-0.20,0.12] | *.61* |  |
| Negative Parenting | 0.05 | [-0.08,0.18] | *.46* | 0.09 | [-0.05,0.24] | *.22* | 0.15 | [0.01,0.29] | *.03* | 0.20 | [0.04,0.36] | *.01* | -0.16 | [-0.33,0.00] | *.06* | -0.00 | [-0.15,0.15] | *.98* |  |
| %Good commands | 0.08 | [-0.03,0.19] | *.16* | 0.10 | [-0.03,0.22] | *.13* | -0.06 | [-0.17,0.04] | *.24* | -0.11 | [-0.27,0.04] | *.16* | 0.13 | [-0.04,0.30] | *.14* | -0.05 | [-0.20,0.10] | *.54* |  |
| %Complied commands | -0.01 | [-0.12,0.11] | *.89* | -0.07 | [-0.20,0.06] | *.27* | 0.05 | [-0.10,0.19] | *.54* | -0.08 | [-0.21,0.05] | *.23* | -0.05 | [-0.20,0.10] | *.50* | 0.22 | [0.07,0.37] | *.00* |  |

***Note:*** *1)* **p* < .05; ***p* < .01; ****p* < .001; 2) The CDI dose × PDI dose interaction effect on Positive Parenting is shown here; the interaction effect on other outcomes also tested but omitted due to non-significance; 3) The β coefficients are unstandardized. Because the outcomes and pretreatment scores were standardized before entry into the model, the unstandardized coefficients can be interpreted as effect sizes—that is, the expected change in the outcome (in standard deviation units) for a one-unit change in the predictor.

Table S2. Results from the Alternative Mediation Model with PCIT Dose Defined as CDI Dose + PDI Dose


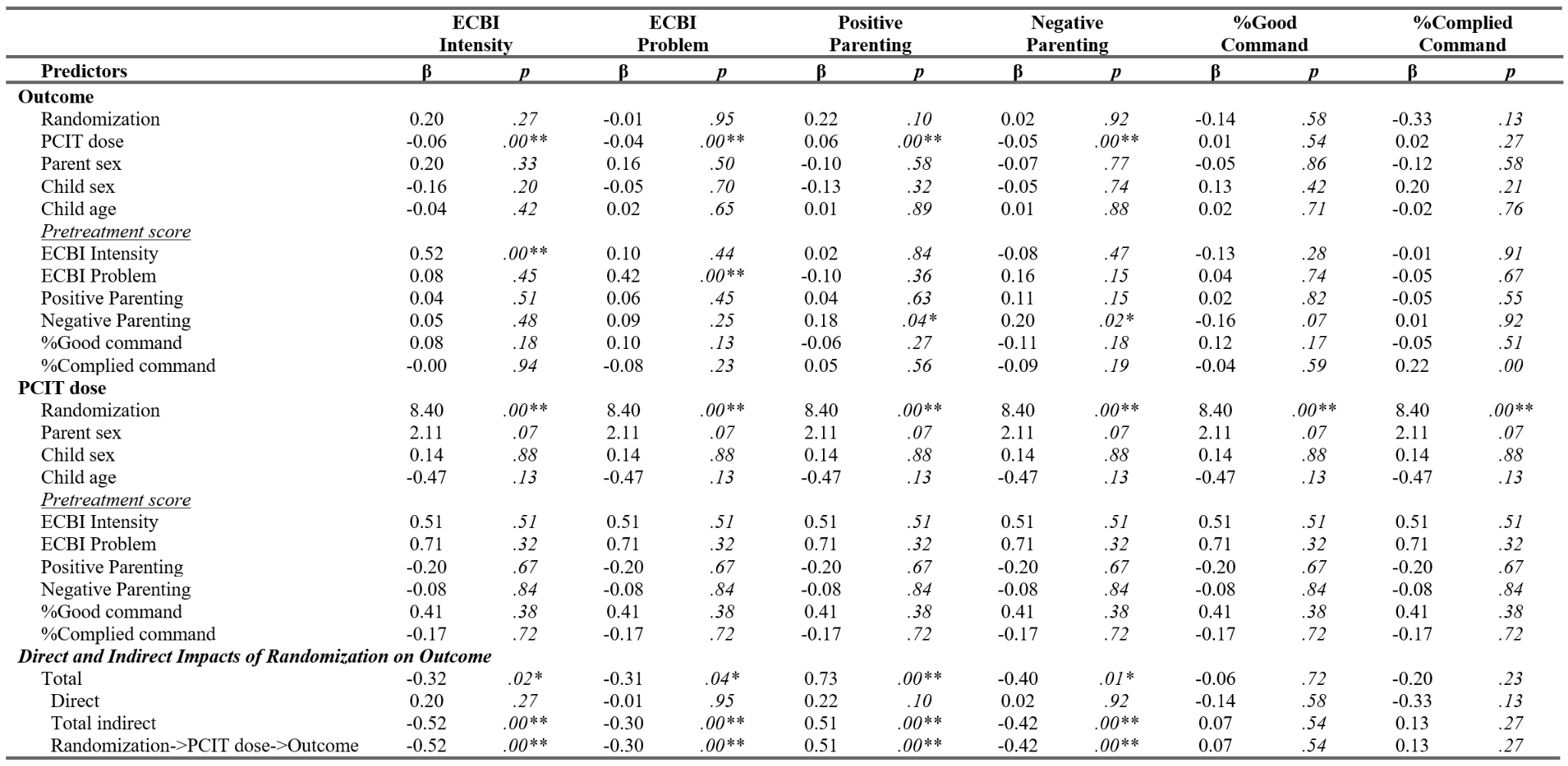


***Note:*** *1)* **p* < .05; ***p* < .01; ****p* < .001; 2) the outcomes and pretreatment scores were standardized before entry into the model


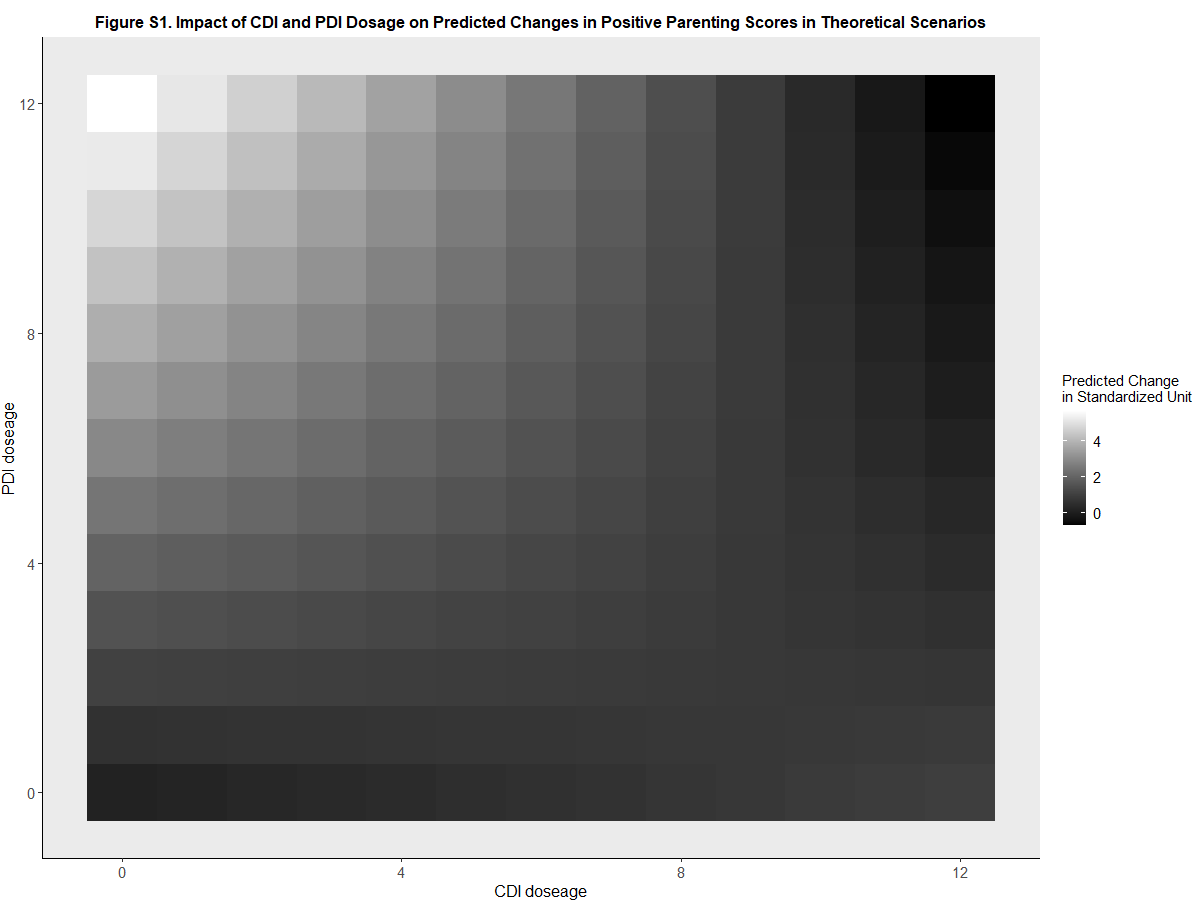


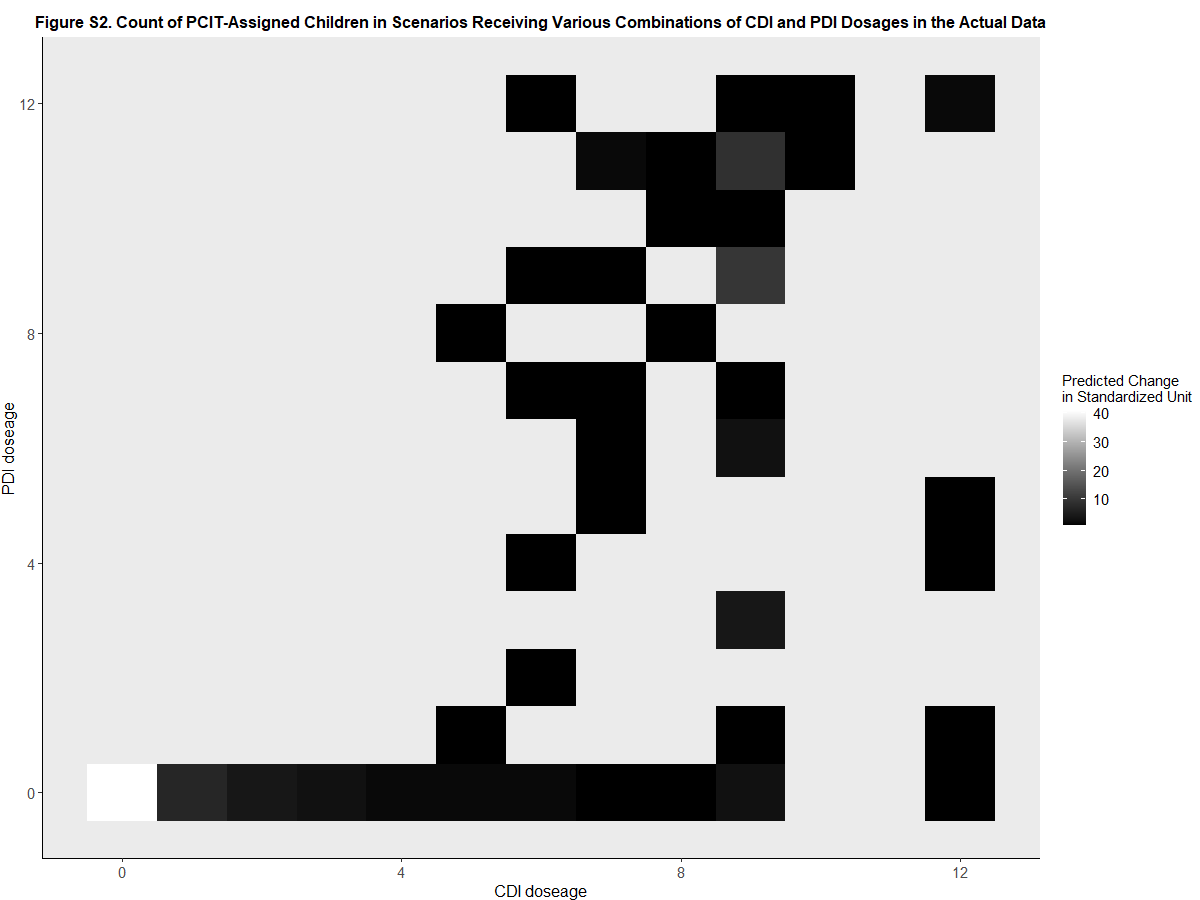


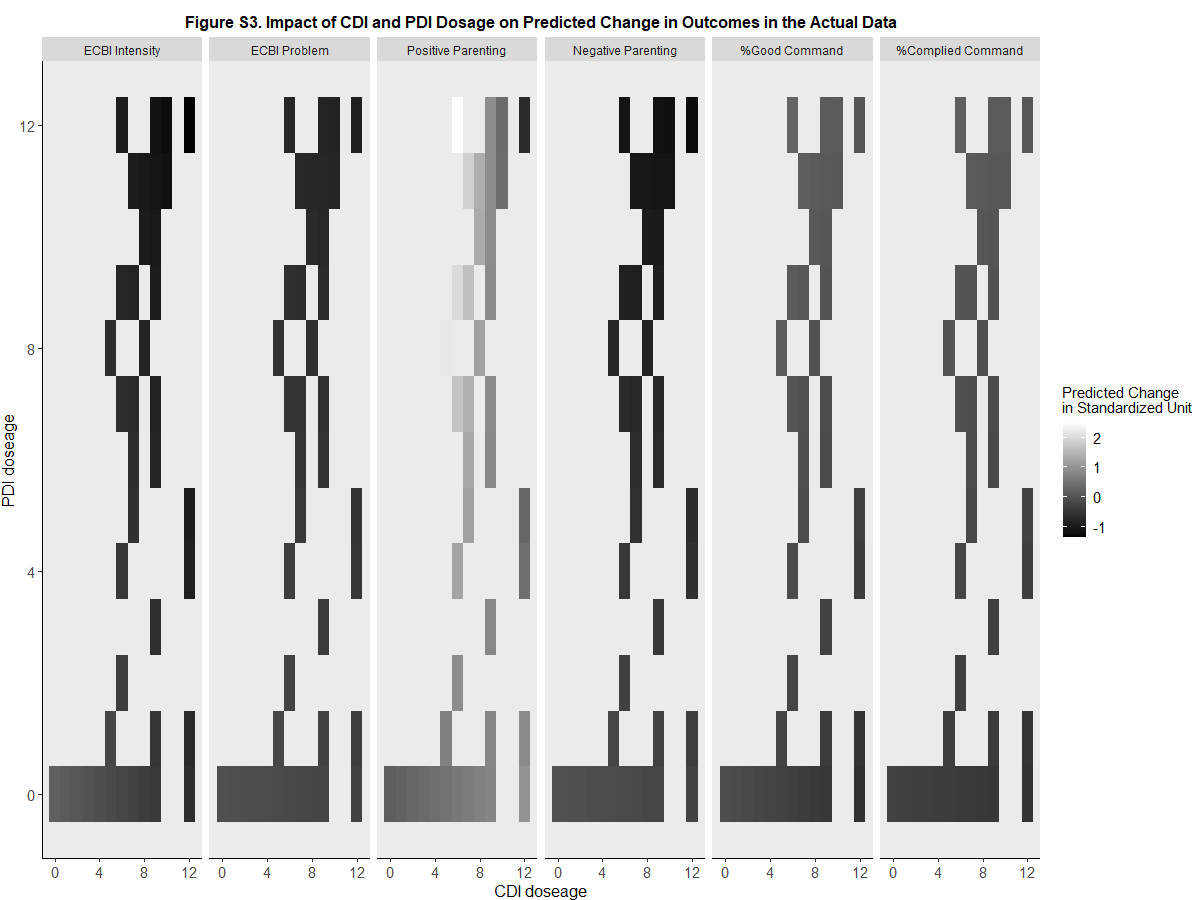

Supplement: Supplementary file 1 — Table S1. Dose–response relationship: results from multiple linear regression. Table S2. Results from the alternative mediation model with PCIT dose defined as CDI dose + PDI dose. Figure S1. Impact of CDI and PDI dosage on predicted changes in positive parenting scores in theoretical scenarios. Figure S2. Count of PCIT‐assigned children in scenarios receiving various combinations of CDI and PDI dosages in the actual data. Figure S3. Impact of CDI and PDI dosage on predicted change in outcomes in the actual data. [file JCPP-67-1224-s001.docx]
